# Hair Specifics

**Published:** 1869-08

**Authors:** 


// HALL'S
### JOURNAL OF HEALTH.

OUR LEGITIMATE SCOPE IS ALMOST BOUNDLESS: FOR WHATEVER BEGETS PLEASURABLE AND HARMLESS FEELINGS, PROMOTES HEALTH: AND WHATEVER INDUCES DISAGREEABLE SENSATIONS ENGENDERS DISEASE.

*We aim to show how Disease may be avoided, and that it is best, when sickness comes, to take no Medicine without consulting an educated Physician.*

VOL. XVI. AUGUST, 1869. No. 8.

### HAIR SPECIFICS.

LET them alone. The whole of them are a cheat There is not one single exception under the sun. A "*specific*" in medicine, is a term which implies certainty of effect. Hair falls out from the want of nutriment. It dies, just as a blade of grass dies in soil where there is no moisture. This want of nutriment is functional or organic. The mechanism which supplies it, the apparatus, is there to make it; but it is out of order, and makes it imperfectly; so the hair being imperfectly nourished, is dry, scant, or a mere furze, according to the degree of the defective nourishment—that is "*Functional Baldness*" and can be remedied radically and permanently in only one way, and that is by taking means to improve the general health.

"ORGANIC" *Baldness* is when the defect of nutriment arises from the destruction of the apparatus which made it; there is no machine there. Under such circumstances nothing short of the power which made man first, can make that hair grow again.

When the scalp is in any part bare of hair, and shiny, or glistening, that is *organic baldness*, and there is no remedy. If there is not that shining, glistening appearance, but a multitude of very small hairs, causing a "*furziness*" over the scalp, that is "functional" baldness; and two things are to be done. Keep the scalp clean with soap-suds—that is a "balm of a thousand flowers," flavored; and more specially, and principally, seek to improve your general health, by eating plain, substantial food, at three regular times a day, and by spending three or four hours, between meals, in moderate exercise in the open air, in some engrossing employment.

As to men, we say, when the hair begins to fall out, the best plan is, to have it cut short, give it a good brushing with a moderately stiff brush, while the hair is dry, then wash it well with warm soap-suds, then rub into the scalp, about the roots of the hair, a little bay rum, or brandy, or camphor water. Do these things twice a month, but the brushing of the scalp may be profitably done twice a week. Dampen the hair with water every time the toilet is made. Nothing ever made is better for the hair than pure soft water, if the scalp is kept clean in the way we have named.

The use of oils, or pomatums, or grease of bears, pigs, geese, or anything else, is ruinous to the hair of man or woman. We consider it a filthy practice, almost universal though it be, for it gathers dust and dirt, and soils whatever it touches. Nothing but pure soft water should ever be allowed on the heads of our children. It is a different practice that robs our women of their most beautiful ornament, long before their prime. The hair of our daughters should be kept within two inches, until their twelfth year.

